# Exploring static and dynamic relationships between burden of disease and research funding in the United States

**DOI:** 10.1186/s12961-022-00837-y

**Published:** 2022-06-03

**Authors:** Alok Nimgaonkar, Anisa Y. Mughal, Hakon Heimer, Vishwajit Nimgaonkar, Dede Greenstein, Alexandra Wright

**Affiliations:** 1 Tufts University School of Medicine, Boston, MA United States of America; 2 University of Pittsburgh, School of Medicine, Pittsburgh, PA United States of America; 3 University of Copenhagen, København, N., Copenhagen, Denmark; 4 Department of Psychiatry, University of Pittsburgh School of Medicine, Pittsburgh, PA United States of America; 5 Department of Human Genetics, University of Pittsburgh, Graduate School of Public Health, Pittsburgh, PA United States of America; 6 Experimental Therapeutics Branch, National Institute of Mental Health, Bethesda, MD United States of America; 7 California State University, Los Angeles, 5151 State University Dr, Los Angeles, CA United States of America

**Keywords:** NIH, Global burden of disease, Mental health, Funding

## Abstract

**Background:**

The relationship between burden of disease and research funding has been examined cross-sectionally, but temporal patterns have not been investigated. It is logical to assume that temporal improvements in disability-adjusted life-years (DALYs) reflect benefits from research funding; such assumptions are tempered by an unknown lag time for emergence of benefits from research.

**Methods:**

We studied National Institutes of Health (NIH) research fund allocations and United States DALY estimates for overlapping disease categories (matched disease categories, MDC, *N* = 38). Using a general linear model, we separately analysed DALYs for MDCs in 2017 in relation to NIH research allocations in 2017 and 2007. We also examined how changes in DALYs were related to cumulative NIH research funding (2006–2017). After regressing DALY change on summed funding, we obtained model residuals as estimates of the discrepancy for each MDC between observed and expected change in burden, given funding.

**Results:**

In 2017, there was a positive association between NIH research fund allocations and DALYs for the same year (*F*_1,36_ = 16.087, *p* = 0.0002921; slope = 0.35020; model *R*^2^ = 0.3088), suggesting proportionate allocation. There was a positive association between 2017 DALYs and 2007 NIH research allocation, implying a beneficial impact of research (*F*_1,36_ = 15.754, *p* = 0.0003; slope = 0.8845; model *R*^2^ = 0.3044). In contrast, there was a nonsignificant association between summed NIH funding and percent change in DALYs over 2006–2017 (*F*_1,36_ = 0.199; *p* = 0.65; beta coefficient = −1.144). When MDCs were ordered based on residuals, HIV/AIDS ranked first. Mental, neurologic or substance abuse (MNS) disorders comprised most residuals in the lower half.

**Conclusions:**

NIH fund allocation is proportional to DALYs for MDCs. Temporal changes in DALYs vary by MDCs, but they are not significantly related to cumulative research outlays. Further analysis of temporal changes in DALYs could help to inform research outlays for MDCs and to study the impact of research.

**Supplementary Information:**

The online version contains supplementary material available at 10.1186/s12961-022-00837-y.

## Background

The National Institutes of Health (NIH) is the largest publicly funded source for healthcare research worldwide [1]. NIH sets funding priorities based on a dynamic balance between public health needs, scientific opportunities, portfolio balance and budgetary considerations, among other factors [2]. The process by which NIH sets priorities is both flexible (to maximize momentum from new developments) and inclusive of voices from various communities (i.e. scientific experts, United States Congress, patients, NIH officials), while still allowing for a high proportion of investigator-initiated research. Even if the burden of individual diseases in the United States influences research funding allocations, a one-to-one correspondence cannot be expected. Previous studies have indeed found this to be the case. For example, in 1999, disease burden as measured by disability-adjusted life-years (DALYs) accounted for approximately 39% of the variation in NIH funding, falling to 33% in 2006 [3, 4].

How disease burden changes in relation to research outlays is an important consideration for public health policy that has not been explored extensively. This is not surprising, given difficulties in harmonizing measures of burden or funding over time. Whereas early NIH funding data provided total annual funding levels, more recent (2006 to 2017) individual disease-specific data are now available through the Research, Condition, and Disease Categorization (RCDC) system [32]. Many of the illnesses listed in the RCDC are also included in the Global Burden of Disease (GBD) study, which until recently did not have measures that could be considered comparable over time. Still, the temporal relationship between changes in burden and funding has not been analysed. Estimates of this relationship could be especially useful when analysed in relation to the other factors that contribute to NIH research allocation decisions. The “time lag” between research initiatives and tangible clinical benefits is another vexing aspect of this research. Although it is reasonable to anticipate a delay after initiation of research into a disorder, it is uncertain whether and when benefits accrue; it is thus difficult to model the time lag aspect of research.

For the current analyses, our aim was twofold. First, we aimed to provide an updated estimate of the burden/funding relationship using the most recent GBD reports cross-sectionally, a type of analysis that we call “cross-sectional” or “static” correlational analysis. We used DALYs to estimate the burden of disease, as this variable encompasses both morbidity and mortality, enabling diseases with high mortality and diseases with high morbidity to be analysed together. As such, DALYs are popular measures of disease burden [5, 6]. Based on previous studies [3, 4], we hypothesized a positive association between NIH funding and DALYs. We fulfilled the first aim of the work, namely cross-sectional correlations, by elucidating a correlation between research funding in 2017 and DALYs in 2017. Our second aim was to examine the “temporal” or “dynamic” change in DALYs in relation to research funding. Whereas we found a significant positive association between research funding in 2007 and DALYs in 2017 (assuming a 10-year time lag), we did not find a significant relationship between NIH summed funding and percent change in DALYs over time.

## Methods

### Data collection

#### NIH research funding

All data were obtained from NIH budgetary spending data and the GBD Results Tool. NIH research funding for specific disorders is generated by the RCDC system and is available on the NIH Research Portfolio Online Reporting Tools (RePORT) website [32].

DALYs The Institute of Health Metrics and Evaluation (IHME) maintains and updates data pertaining to the GBD, including DALYs, in their results tool [33]. DALY values were standardized for age.

### Data processing

#### Measuring changes over time

For each MDC, we summed NIH funding for the period 2006–2017 and calculated percent change in DALYs from 2006 to 2017 (2017 DALY − 2006 DALY / 2017 DALY).

#### Merged disease categories

Prior to aligning NIH and GBD categories, NIH categories were inspected for consistency in nomenclature across time and, if necessary, merged to align with GBD categories (Additional file 1). We identified disease categories with complete classification overlap between the NIH and GBD data sources, excluding broad categories in one dataset that overlapped with narrower categories in the other, or vice versa (e.g. cardiovascular disease. Notably, funding for “Alzheimer’s Disease” (RCDC) from 2006–2014 and “Alzheimer’s Disease including Alzheimer’s Disease Related Dementias (AD/ADRD) 2/” for 2015–2017 were used to match with “Alzheimer’s disease and other dementias” (GBD) to ensure consistency across years. Some disease categories were excluded because relevant data were missing from some years, namely anxiety disorders, eating disorders, hepatitis, migraines and pancreatic cancer. Other disease categories were discarded because of insufficient GBD data for the United States or because we aimed to maintain consistency with prior reports [3]: digestive diseases, Down syndrome, endometriosis, otitis media, psoriasis, sudden infant death syndrome. A list of 38 matched disease categories (MDCs; Additional file 1: Table S1) were thus generated and used for combined analyses of NIH and GBD data.

### Data analysis

DALY and funding variables were log-transformed for all analyses, because both variables approximated a log-normal distribution. To assess the contemporaneous/cross-sectional relationship between burden and funding, we regressed 2017 age-standardized DALYs on 2017 NIH MDC funding, and to assess the 10-year time-lagged relationship between funding and subsequent DALYs, we regressed 2017 age-standardized DALYs on 2007 NIH MDC funding. To examine the temporal relationship between changes in DALYs and NIH funding, we regressed DALY percent change on summed NIH funding for individual MDCs from 2006–2017. For each regression, we examined plots of residual distributions as well as leverage and influence plots. We also conducted sensitivity analyses to determine whether and how the observed results were altered by removing possible influential data points.

## Results

### Cross-sectional analyses

**Fig. 1:**
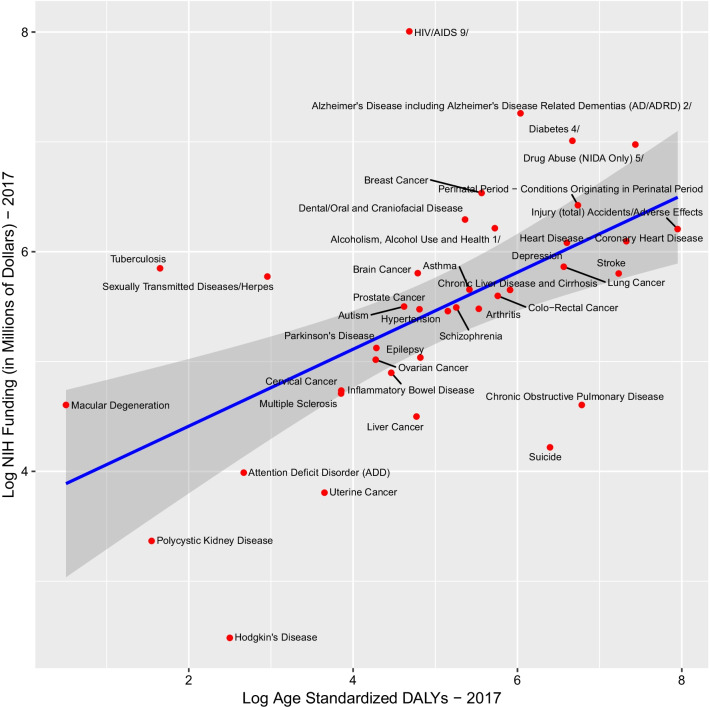
Log 2017 GBD DALYs and Log 2017 NIH funding. GBD estimates for the United States for individual diseases/disease groups were plotted against the respective NIH funds. All data were log-transformed

**Fig. 2:**
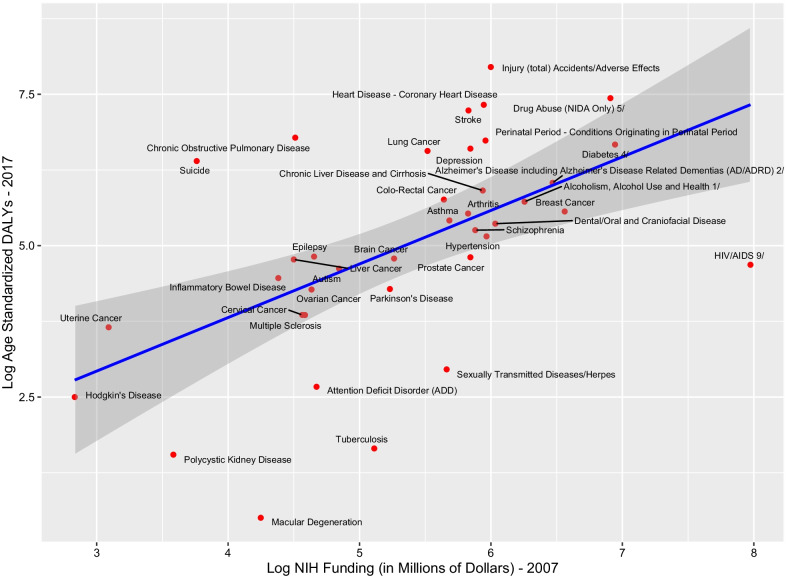
Log 2007 NIH funding and Log 2017 GBD DALYs. NIH funds for individual diseases/disease groups were plotted against the respective GBD estimates for the United States. All data were log-transformed

NIH research expenditure in 2017 was positively related to 2017 DALYs for MDCs (*F*_1,36_ = 16.087, *p* = 0.0002921; slope = 0.35020; model *R*^2^ = 0.3088), implying a modest but statistically significant linear relationship (Fig. 1). This relationship remained significant after removal of outliers (Hodgkin’s disease or HIV/AIDS; data not shown). NIH research expenditures in 2007 were also positively related to 2017 DALYs for MDCs, although this relationship was weaker than the contemporaneous burden/funding relationship with DALYs (*F*_1,36_ = 15.754, *p* = 0.0003; slope = 0.8845; model *R*^2^ = 0.3044) (Fig. 2). This relationship remained significant after removal of outliers (Hodgkin’s disease or HIV/AIDS; data not shown).

### Temporal analyses

**Fig. 3:**
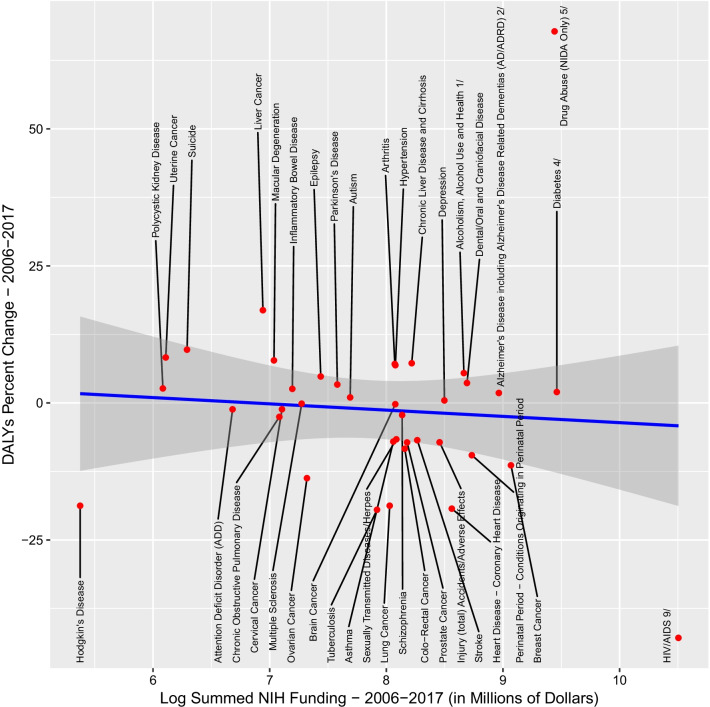
Overall percent change in DALYs for the period 2006–2017 and log-transformed cumulative summed NIH funding 2006–2017. Percent change in DALYs from 2006 to 2017 for individual diseases and disorders was plotted against cumulative NIH funding for the same diseases/disorders during 2006–2017 (in millions of US dollars, log-transformed values used for clarity)

**Fig. 4:**
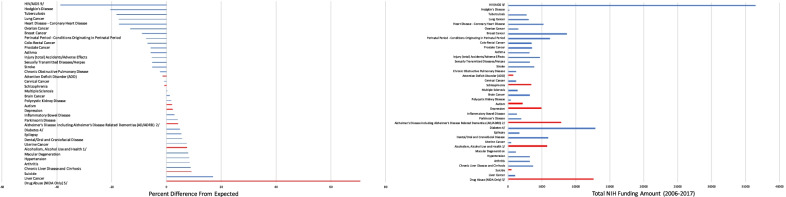
Difference in overall percent change in DALYs from that expected for individually matched diseases. Left panel: Residuals from a model with percent change in DALYs (2006–2017) regressed on log-transformed NIH funding summed over the same time. Right panel: Summed NIH funding amounts (2006–2017, not log-transformed, in millions of US dollars). Red bars indicate psychiatric and drug abuse disorders; blue bars indicate all other diseases/disorders

We next analysed changes in DALYs from 2006 to 2017 in relation to cumulative research outlays for the same period. Unlike the analyses relating 2017 DALYs to 2007 research allocations, we did not detect a significant relationship (*F*_1,36_ = 0.199; *p* = 0.65; beta coefficient = −1.144) between summed NIH funding from 2006 to 2017 and percent change in DALYs for this period (Fig. 3). Residuals from this model are estimates of each MDC’s positive or negative deviation from their expected change in burden given the associated funding levels and are plotted in Fig. 4 alongside their total funding (Fig. 4). HIV/AIDS, Hodgkin’s disease and tuberculosis have some of the largest negative residual values. Suicide, liver cancer and drug abuse have some of the highest residuals, indicating that DALYs increased more than expected relative to funding. MNS disorders are overrepresented in the lower half of this list (10/19 in the lower half versus 4/19 in the upper half, chi-square = 2.83, *p* = 0.09 with Yates’s correction).

## Discussion

It is very important to note that all our analyses are correlative in nature, so causation cannot and should not be inferred between research funding and DALYs. Our first goal was to evaluate NIH research fund allocation in relation to disease burden, which we dubbed “cross-sectional” correlational analyses. The significant correlation between research fund allocation in 2017 and DALYs in 2017 suggests that NIH fund allocations for diseases are proportional to their burden on United States populations. Our results are consistent with earlier studies of NIH funding outlays, as well as those of different health systems [3, 4]. For example, analyses of health systems in Norway, Canada, China and South Korea have noted similar correlations between funding and DALYs [30–32]. A 2012 analysis of NIH funds in relation to DALYs found correlations similar to our analyses (previous study, *R*^2^ = 0.26 vs 0.29 in the present study) [3–7]. These investigators noted disproportionately higher investment in cancer and HIV/AIDS research, as well as diabetes mellitus and perinatal disorders, relative to other diseases. Earlier analyses also indicated that NIH resource allocations are significantly correlated with frequency of hospitalization and mortality [4]. In sum, the published results reflect the stated role of disease burden in NIH research allocation decisions [2–4, 7]. The rate of industry-sponsored research for basic research is declining, with greater emphasis on medical devices, bioengineered drugs and late-stage clinical trials [7]. In the face of declining resources, more stringent and fine-grained analyses in relation to research allocations are needed; the present emphasis on cross-sectional patterns serves as a promising start, but is likely insufficient to provide the necessary information.

There is likely a time lag between research funding and any meaningful effects on DALYs, but it is very difficult to estimate the lag precisely; furthermore, the lag duration is likely to differ among different diseases. Mindful of these concerns, and assuming a 10-year time lag, we examined 2007 NIH research outlays in relation to 2017 DALYs. We found a significant positive relationship. Still, these analyses should be interpreted with caution, as it is difficult to model the appropriate lag time between research fund allocation and impact on DALYs. Such analyses are also simplistic, implying a causal relationship in a highly complex scenario. For example, social and cultural factors likely have a substantial impact on DALYs, particularly for psychiatric disorders. Temporal change in disease burden could represent a more sensitive index for analyses in relation to research resources than cross-sectional point-in-time indices, as temporal changes can reflect longer-term trends. Therefore, our additional goal—our “temporal” analyses—was to study associations between NIH research fund allocation as the independent variable and disease burden as an outcome: mindful of the aforementioned time lags, we analysed data over the entire available time span. When DALYs were examined in relation to NIH spending over a 10-year period, no significant linear relationship was noted between the overall percent change in DALYs and summed NIH funding. Whereas approximately half of MDCs were associated with declines in DALYs greater than the predicted levels based on overall correlations (e.g. HIV, tuberculosis), those classified as mental, neurologic or substance abuse (MNS) disorders constituted most categories in the lower half. The greatest increase over time relative to NIH research allocations occurred for HIV, whereas drug abuse disorders ranked the lowest.

The favourable change in HIV disease burden provides an example for emulation. Central to the HIV success story is the availability of resources for research and programmatic efforts. Driven by high mortality rates and high DALYs [8], a concerted research effort for HIV/AIDS was launched in the 1980s and 1990s [7] In fact, NIH spending for HIV/AIDS earned its own separate funding process in the 1980s, as a response to its place as the leading cause of death for people aged 25–44 years in the United States [9]. Indeed, the period from 1995 to 2013 witnessed a reduction in HIV/AIDS deaths per annum from 45 000 to 7000 [10]. While mortality is only one measure, newly diagnosed cases decreased by 25.2% from 2003 to 2013 as well [11]. Much of the reduction in the disease burden due to HIV/AIDS can be attributed to drug development, a direct result of increased research investment [12]. Arguably, the introduction of double-nucleoside therapy and protease inhibitors did the most to lay the foundation for treatment and reduction in mortality during this period [13]. In a large, national surveillance study of HIV, antiretroviral therapies had the greatest effect on morbidity and mortality [14]. Thus, the huge research efforts quickly led to an understanding of HIV and subsequent development of many different antiretroviral therapies [15].

On the other hand, the increase in drug abuse disorder DALYs despite increased NIH research allocation presents a challenge for researchers, research agencies and healthcare providers alike. The increase in DALYs likely stems from the increase in rates of drug overdose-related emergency department visits and hospitalizations that rose astronomically from 2002 to 2012 [16]. Deaths owing to overdose from 1999 to 2016 nearly tripled [17]. The causes are likely multifactorial, with a substantial role for societal factors such as increased prescription of painkillers in myriad circumstances and the availability of counterfeit and new illicit drugs like fentanyl [17]. Correspondingly, access to treatments for opioid use disorders is low; few doctors are trained in providing known treatments for opioid disorders, and those who are often employ inconsistent treatment approaches. Additional logistical barriers such as long wait times and lack of coverage for treatment exacerbate the situation [18]. Nevertheless, resource allocation is beginning to catch up, with government sectors diverting more money towards the opioid crisis [19]. More germane to this study, the NIH’s recent Helping to End Addiction Long-term^®^ (HEAL) project has committed US$ 500 million in research and collaborative efforts to combat the crisis [19]. It will be important in the future to relate research efforts and other programmes jointly to changes in DALYs.

There are several plausible reasons for the relatively small change in DALYs for MNS disorders relative to research investment. First and foremost, the dearth of adequate measurements of changes in psychiatric disease burden limits the accurate measurement of changes. The increased incidence of Alzheimer’s disease could also be a factor. While other diseases have tangible changes that could be estimated using estimates such as DALYs or mortality, measures of symptom severity and improvement at the population level for disorders such as depression are currently unavailable, and datasets that are available lack the sensitivity to detect subtle changes. It should be noted that NIH invests in a broad array of research, but the relative lack of effective available treatments likely contributes substantially to the discrepancy between funding and reduction in DALYs. Even with regard to pharmacological research, the cost, discovery and time may vary significantly. The lack of a significant association between research allocation and change in DALYs could also reflect lower allocations: it could be argued that levels of funding proportionate to those for HIV/AIDS would lead to improvements for these disorders too. Unlike diseases such as HIV/AIDS, psychiatric disorders may also be less tractable to improvement [19]; for example, factors such as societal and individual-level stigma are difficult to change [21, 22]. Furthermore, low treatment-seeking behaviour and undertreatment still remain a large problem even in the United States, and the quality of mental health services for those receiving treatment could also be improved regardless of research fund allocations [23]. There is also a relatively low emphasis on preventive efforts globally [24]. Several proposals outside the domain of research agencies such as the NIH could thus be considered—for example, establishing a global mental health fund to generate interest and investment and efforts to reduce the stigma of mental health disorders [25, 26]. Suicide and liver cancer are also among the categories with the lowest change in burden given the level of funding, indicating that lack of change in DALYs occurs in non-MNS disorders as well.

Some important limitations of this study should be noted. We recognize that numerous factors apart from research funding can and do affect indices of disease burden such as DALYs [27]. Our measures of change, specifically percent change in DALYs and cumulative funding, are relatively “blunt” tools. The latter does not account for inflation; adjusting each year’s totals to current-year dollars arguably addresses this limitation. In addition, we acknowledge that the period of analysis—2006 to 2017—may be too short to expect a significant impact of NIH spending on DALYs. GBD estimates are calculated annually independent of the previous year’s estimates; thus it is difficult to evaluate fine-grained changes over a decade. While this problem could be addressed in the present analyses by incorporating data from intervening years when relating NIH allocations to changes in DALYs, we chose not to do so, because NIH spending underwent substantial changes during the 2008–2017 period, and we thus reasoned that cumulative funding would be a more appropriate approach. Significant reductions in burden of disease are expected to lag research findings even if the research directly alleviates disease burden, but our analyses could not account for a lag factor in burden of disease, as the lag period is likely to differ substantially by type of research investment and by the features of individual diseases. Definitions and reporting on DALYs change periodically; moreover, even though percent change in DALYs is meaningful from a public health perspective, percent changes in DALYs do not perfectly capture an MDC’s fluctuations over time. Further subdivision by type of research investment—for example, preclinical, clinical or translational—could not be analysed in relation to disease burden, as relevant information was not available from the NIH database [28]. We did not account for the contributions of other research funding agencies and research at pharmaceutical companies that could also contribute to changes in DALYs [7, 32]. Many disorders could not be analysed because precisely matched NIH research data and DALYs were unavailable.

It is also important to note that our sole reliance on DALYs to estimate burden of disease does not fully capture the impact of NIH resource allocation. Additional outcome metrics such as success in licensing and marketing of drugs or a so-called research opportunity index could measure returns on NIH investment as well [29]. Research publications have also been used as metrics for research progress, although prior analyses show that, globally, DALYs for individual diseases are negatively correlated with research publications [30].

While the present analyses are related to the United States, we believe that a similar metric could still be relevant to measuring international research output. A research opportunity index, which measures imbalances between the optimal and actual resource allocation for individual diseases and for all diseases in aggregate, has also been proposed as a suitable variable that should be evaluated in future studies [31].

## Conclusions

NIH research investment in 2017 was proportional to 2017 DALYS for MDCs in the United States, consistent with cross-sectional analyses reported in the past; these analyses support the notion that burden imposed by individual diseases/disease groups is given consideration during NIH research allocations. On the other hand, our “temporal” analyses indicated that cumulative NIH investment during 2006–2017 was not significantly correlated with changes in DALYs for selected MDCs over this period. Analysis of residuals from this model showed that improvement in DALYs for diseases like HIV/AIDS, Hodgkin’s disease and tuberculosis was greater than would be expected in relation to NIH research outlays for these diseases. In contrast, changes in DALYs for other diseases—among which MNS disorders predominated—were smaller than would be expected from research outlays for these diseases. The present analyses do not enable causal inferences, so prospective investigations would be informative.

## Supplementary Information

**Additional file 1: Table S1.** GBD and NIH merged disease categories. This file includes categories from the IHME dataset and NIH datasets that were matched and included in the analysis.

## Data Availability

The datasets supporting the conclusions of this article are available in the NIH Research Portfolio Online Reporting (RePORT) website repository [32], and in the Institute of Health Metrics and Evaluation (IHME) [33]. This is a dynamic dataset that is continuously updated and curated.

## Abbreviations

DALYs: Disability-adjusted life-years
GBD: Global burden of disease
BD: Burden of disease
MDC: Matched disease categories
MNS: Mental, neurologic, or substance abuse disorders
RCDC system: Research, Condition, and Disease Categorization system
NIH RePORT website: NIH Research Portfolio Online Reporting website
IHME: Institute of Health Metrics and Evaluation
