# Supplementary material for: Exploring static and dynamic relationships between burden of disease and research funding in the United States
Source: Health Res Policy Syst. 2022 Jun 3;20:60. doi: 10.1186/s12961-022-00837-y (PMC9164716; doi:10.1186/s12961-022-00837-y)
Supplement: Supplementary file 1 — Additional file 1: Table S1. GBD and NIH merged disease categories. This file includes categories from the IHME dataset and NIH datasets that were matched and included in the analysis. [file 12961_2022_837_MOESM1_ESM.pdf]

**Additional File 1:Table S1. GBD and NIH Merged Disease Categories**

| GBD Categories | NIH Research Disease Categories 2006-2007 | NIH Research Disease Categories 2008-2014 | NIH Research Disease Categories 2014-2017 |
|---|---|---|---|
| Age-Related Macular Degeneration | Macular Degeneration | Macular Degeneration | Macular Degeneration |
| Alcohol use disorders | Alcoholism | Alcoholism, Alcohol Use and Health 1/ | Alcoholism, Alcohol Use and Health 1/ |
| Alzheimer's disease and other dementias | Alzheimer’s Disease | Alzheimer's Disease | Alzheimer’s Disease (2014); Alzheimer's Disease including Alzheimer's Disease Related Dementias (AD/ADRD) 2/ (2015-2017) |
| Arthritis_NEW | Arthritis | Arthritis | Arthritis |
| Asthma | Asthma | Asthma | Asthma |
| Attention-deficit/hyperactivity disorder | Attention Deficit Disorder (ADD) | Attention Deficit Disorder (ADD) | Attention Deficit Disorder (ADD) |
| Autism spectrum disorders | Autism | Autism | Autism |
| Brain and nervous system cancer | Brain Cancer | Brain Cancer | Brain Cancer |
| Breast cancer | Breast Cancer | Breast Cancer | Breast Cancer |
| Cervical cancer | Cervical Cancer | Cervical Cancer | Cervical Cancer |
| Chronic obstructive pulmonary disease | Chronic Obstructive Pulmonary Disease | Chronic Obstructive Pulmonary Disease | Chronic Obstructive Pulmonary Disease |
| Cirrhosis and other chronic liver diseases | Chronic Liver Disease and Cirrhosis | Chronic Liver Disease and Cirrhosis | Chronic Liver Disease and Cirrhosis |
| Colon and rectum cancer | Colo-Rectal Cancer | Colo-Rectal Cancer | Colo-Rectal Cancer |
| Depressive disorders | Depression | Depression | Depression |
| Diabetes mellitus | Diabetes | Diabetes 4/ | Diabetes 4/ |
| Drug use disorders | Drug Abuse (NIDA only) | Drug Abuse (NIDA Only) 5/ | Drug Abuse (NIDA Only) 5/ |
| Epilepsy | Epilepsy | Epilepsy | Epilepsy |
| HIV/AIDS | HIV/AIDS114 | HIV/AIDS 9/ | HIV/AIDS 9/ |
| Hypertensive heart disease | Hypertension | Hypertension | Hypertension |
| Hodgkin Lymphoma | Hodgkin's Disease | Hodgkin's Disease | Hodgkin's Disease |
| Inflammatory Bowel Disease | Inflammatory Bowel Disease | Inflammatory Bowel Disease | Inflammatory Bowel Disease |
| Injuries | Injury (total) Accidents/Adverse Effects | Injury (total) Accidents/Adverse Effects | Injury (total) Accidents/Adverse Effects |
| Ischemic heart disease | Heart Disease: Coronary Heart Disease | Heart Disease - Coronary Heart Disease | Heart Disease - Coronary Heart Disease |
| Liver cancer | Liver Cancer | Liver Cancer | Liver Cancer |
| Multiple sclerosis | Multiple Sclerosis | Multiple Sclerosis | Multiple Sclerosis |
| Neonatal disorders | Perinatal Period, Conditions Originating in Perinatal Period | Perinatal Period - Conditions Originating in Perinatal Period | Perinatal Period - Conditions Originating in Perinatal Period |
| Oral disorders | Dental/Oral and Craniofacial Disease | Dental/Oral and Craniofacial Disease | Dental/Oral and Craniofacial Disease |
| Ovarian cancer | Ovarian Cancer | Ovarian Cancer | Ovarian Cancer |
| Parkinson's disease | Parkinson’s Disease | Parkinson's Disease | Parkinson's Disease |
| Polycystic Ovarian Syndrome | Polycystic Kidney Disease | Polycystic Kidney Disease | Polycystic Kidney Disease |
| Prostate cancer | Prostate Cancer | Prostate Cancer | Prostate Cancer |
| Schizophrenia | Schizophrenia | Schizophrenia | Schizophrenia |
| Self-harm | Suicide | Suicide | Suicide |
| Sexually transmitted infections excluding HIV | Sexually Transmitted Diseases/Herpes | Sexually Transmitted Diseases/Herpes | Sexually Transmitted Diseases/Herpes |
| Stroke_NEW | Stroke | Stroke | Stroke |
| Tracheal, bronchus, and lung cancer | Lung Cancer | Lung Cancer | Lung Cancer |
| Tuberculosis | Tuberculosis | Tuberculosis | Tuberculosis |
| Uterine cancer | Uterine Cancer | Uterine Cancer | Uterine Cancer |

## Individual Disease Categories Collapsed to match GBD or NIH

**Arthritis.** ‘Osteoarthritis’ and ‘Rheumatoid arthritis’ (GBD) were summed to match “Arthritis” (RCDC)
**Stroke**.‘Stroke’ and ‘Ischemic Stroke’ and ‘intracerebral hemorrhage’ and ‘subarachnoid hemorrhage’ (GBD) were summed to match ‘Stroke’ (RCDC)

**Alzheimer’s Disease and Related Dementias (RCDC).** ‘Alzheimer's Disease’ (RCDC 2006-20014) and ‘Alzheimer's Disease including Alzheimer's Disease Related Dementias (AD/ADRD) 2/’ (RCDC 2015-2017) were used to match ‘Alzheimer’s disease and other Dementias’ (GBD)
